# Facet joint syndrome: from diagnosis to interventional management

**DOI:** 10.1007/s13244-018-0638-x

**Published:** 2018-08-08

**Authors:** Romain Perolat, Adrian Kastler, Benjamin Nicot, Jean-Michel Pellat, Florence Tahon, Arnaud Attye, Olivier Heck, Kamel Boubagra, Sylvie Grand, Alexandre Krainik

**Affiliations:** 1 Clinique Universitaire de Neuroradiologie, Centre Hospitalier Universitaire A Michallon, Grenoble, France; 2 Clinique Universitaire de Radiologie et Imagerie Médicale, Centre Hospitalier Universitaire, A. Michallon, BP 217, 38043 Grenoble Cedex 9, France; 3 Service de Neurochirurgie, Centre Hospitalier Universitaire A. Michallon, Grenoble, France; 4 Centre d’évaluation et du traitement de la douleur, Groupe hospitalier mutualiste, Grenoble, France

**Keywords:** Low back pain, Facet joint, Block, Neurolysis, Radiofrequency, Cryoablation

## Abstract

**Abstract:**

Low back pain (LBP) is the most common pain syndrome, and is an enormous burden and cost generator for society. Lumbar facet joints (FJ) constitute a common source of pain, accounting for 15–45% of LBP. Facet joint degenerative osteoarthritis is the most frequent form of facet joint pain. History and physical examination may suggest but not confirm facet joint syndrome. Although imaging (radiographs, MRI, CT, SPECT) for back pain syndrome is very commonly performed, there are no effective correlations between clinical symptoms and degenerative spinal changes. Diagnostic positive facet joint block can indicate facet joints as the source of chronic spinal pain. These patients may benefit from specific interventions to eliminate facet joint pain such as neurolysis, by radiofrequency or cryoablation. The purpose of this review is to describe the anatomy, epidemiology, clinical presentation, and radiologic findings of facet joint syndrome. Specific interventional facet joint management will also be described in detail.

**Teaching points:**

*• Lumbar facet joints constitute a common source of pain accounting of 15–45%.*

*• Facet arthrosis is the most frequent form of facet pathology.*

*• There are no effective correlations between clinical symptoms, physical examination and degenerative spinal changes.*

*• Diagnostic positive facet joint block can indicate facet joints as the source of pain.*

*• After selection processing, patients may benefit from facet joint neurolysis, notably by radiofrequency or cryoablation.*

## Introduction

Chronic low back pain is one of the most common pain syndromes and represents an enormous burden and cost generator for society [1]. Lumbar facet joints (FJs) constitute a common source of pain and remain a misunderstood, misdiagnosed and improperly treated pathology [2]. Facet osteoarthritis is the most frequent form of facet pathology [3]. Although imaging for back pain syndrome is very common (radiographs, MRI, CT, SPECT), there is no effective correlation between clinical symptoms and degenerative spinal changes [4], with some imaging findings that may, in specific cases, appear irrelevant to the clinical setting. Clinical facet joint syndrome is defined as a unilateral or bilateral back pain radiating to one or both buttocks, sides of the groin, and thighs, and stopping above the knee [5]. However, in some cases, patients’ symptoms in the setting of low back pain may lack specificity, as facet joints may mimic the pain caused by herniated discs or compressed roots. History and physical examination may suggest, but not confirm FJs as the source of pain [6]. A diagnostic positive facet joint block can indicate facet joints as the source of chronic spinal pain [7], but the rate of false positives remains high. After conservative management failure, these patients may benefit from articular steroid injections [8] and/or specific interventions to eliminate facet joint pain such as neurolysis [9]. Radiologists play an important role in the management of back pain, as imaging of spinal disorders has become one of the keys to better patient management. Moreover, interventional radiology has become a keystone of facet joint management, as both a diagnostic and a therapeutic tool. Therefore, this review aims to provide the radiologist with specific information on facet joint epidemiology, anatomy and physiopathology, and its implication in chronic low back pain. Furthermore, the authors describe the essential knowledge of facet joint imaging modalities along with a detailed description of existing interventional management.

## Epidemiology

Chronic and recurrent pain has been defined as a specific health care problem and is considered a disease in its own right [10]. A recent survey showed a high prevalence of chronic pain of moderate to severe intensity in adult Europeans, affecting the quality of their social and working lives and is therefore a major health care problem in Europe [1]. Low back pain (LBP) is one of the most common pain syndromes and is an enormous burden and cost generator for society. The high health care costs may be attributed to multiple factors, including lack of an accurate diagnosis [2], imaging overuse, unwarranted surgery and working stoppages. LBP is responsible for functional limitations and causes difficulty in performing common daily life tasks, especially among the elderly [11]. Therefore, LBP is the most expensive disease in industrialized countries, as has been reported in Germany at a total cost of 48.960 billion euros per year [12]. In the USA, the prevalence of LBP is reportedly between 15 and 45% according to cross-sectional studies [13]. Most spinal structures may be source of LBP, including intervertebral discs, FJs, sacroiliac joints and nerve roots, and may be accessible to diagnostic tests including imaging. Some disorders, particularly disc-related impairments, are reasonably easily diagnosed and lead to definitive treatments. However, discogenic LBP without disc herniation, lumbar FJ, and sacroiliac joint pain are difficult to diagnose with imaging only [2]. The literature focuses on intervertebral discs as the source of LBP; however, FJ pain also seems to play a major role in generating LBP [8]. Among LBP patients, there are wide discrepancies in the reported prevalence of FJ pain. Reviews implicate FJs as the primary pain generator in 10–15% of young adult patients with chronic LBP and higher in older populations (15% among injured workers, 40% in older population without pre-existing trauma, 45% in a more heterogeneous population) [14]. Controlled diagnostic studies have shown a prevalence of lumbar FJ pain of 27–40% in patients with chronic LBP [15].

## Anatomy of facet joints (FJs)

**Fig. 1:**
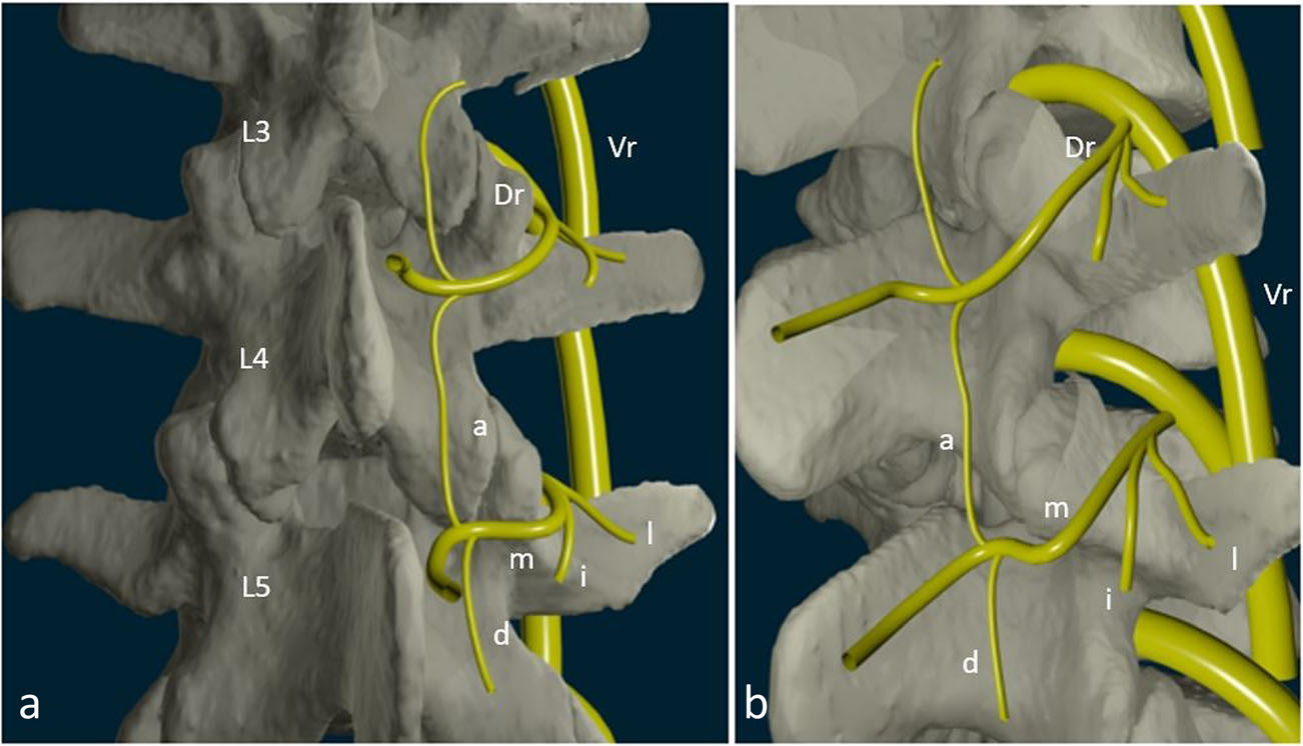
**Innervation of facet joints (L3–4, L4–5 levels)**. Vr: ventral ramus. Dr: Dorsal ramus. m: medial branch. i: intermediate branch. l: lateral branch a: ascending branch. d: descending branch. Posterior (**a**) and posterolateral (**b**) view of the lumbar spine

Each spinal segment consists of an intervertebral disc and posterior paired synovial joints (FJ) comprising a “three-joint complex”, where each component influences the other two, with degenerative changes in one joint affecting the biomechanics of the whole complex. FJs constitute the posterolateral articulation connecting the posterior arch between vertebral levels. They are a paired and diarthrodial joint and are the only synovial joints in the spine, with hyaline cartilage overlying subchondral bone, a synovial membrane and a joint capsule [16]. The joint space presents capacity of 1–2 mL [15]. Each joint comprises an anteriorly and laterally facing inferior articular process from the superior vertebral level and reciprocally a larger, posteriorly and medially facing concave superior, articular process from the inferior vertebral level. Morphological variations may occur within the lumbar spine, as lumbosacral transitional vertebra (defined as either sacralization of the lowest lumbar segment or lumbarization of the most superior sacral segment of the spine). They are common in the general population, with a reported prevalence of 4–30%, with varying morphology, ranging from broadened transverse processes to complete fusion (Castellvi classification) [17]. Knowledge of such variations is essential to avoid an intervention at an incorrect level (see below). The axial morphology of the lumbar FJ from L3 to S1 has been shown to assume a gradually more coronal orientation compared to proximal lumbar levels, with a maximal transverse articular dimension to the distal end. The orientation of the lumbar FJ in the sagittal plane allows for a greater range of flexion motion and prevents gross rotatory instability [18]. Facet joint tropism has been defined as an asymmetry between right and left FJ angles, with one joint having more of a sagittal orientation than the other. Some studies found a relationship among patients who had a symptomatic disc herniation or degenerative spondylolisthesis at L4–5 or L5–1 levels, and an increased severity of facet joint tropism [19]. FJs play an important role in load transmission, providing a posterior load bearing helper, stabilizing the motion segment in flexion and extension. They are also involved in the mechanism of rotational kinematics by restricting the axial rotation [20]. This is achieved through a collagenous tissue of the fibrous capsule layed in a transverse plane providing resistance to flexion motions [16]. Because of their high level of mobility and the important forces influencing in the lumbar area, they can develop significant degenerative changes and be a potential source of pain [21]. The capsule of the FJs, subchondral bone and synovium are richly innervated with nociceptive and autonomic nerve fibres [22]. Substance P nerve fibres have been identified in subchondral bone in degenerative lumbar FJ [23]. Inflammatory mediators such as prostaglandins and cytokines (IL6, TNFα) have been found in cases of degenerative disorders [24]. This partly explains the origin of LBP in case of FJ degeneration. Bogduk et al. [25] were the first to describe three ramifications of the dorsal branch (medial, intermediate and lateral branch) of the spinal nerve, which spread within the dorsal muscles (Fig. 1). From L1 to L4 segments, each lumbar FJ is innervated by the medial branch of the dorsal rami (MBDR). It emerges from the inter-transversal ligament. This branch crosses the superior margin of the medial termination of the transverse process, passing through the caudal root of the superior articulate process (SAP) one level below (i.e. the MBDR of L4 level passes around the SAP of L5). At this level the nerve runs downwards, and is fixed by the mamillo-accessory ligament (MAL). It then enters the multifidus muscle [26]. Intermediate and lateral branches emerge from the dorsal ramus, they run caudally and laterally and enter respectively the longissimus and iliocostalis muscles. Each joint is innervated by a dual supply from the medial branch at the same level and the level above [27] with ascending and descending branches. The L5 segment has a different distribution of the branches, which should be considered in FJ denervation [25]. First of all, the dorsal ramus is longer; it emerges dorsally and in the inferior region on top of the sacrum wing, along the groove formed between the ala of the sacrum at the root of the S1 SAP, and runs near the inferior portion of the articular process. The nerve then ramifies in an intermediate and a medial branch. There is no lateral branch; the MBDR lies caudally to the process, running into a fibrous tissue equivalent to the MAL, with communicating branches with the S1 dorsal ramus. Four factors were described for an anatomical structure to be deemed a cause of back pain: a nerve supply to the structure, the ability of the structure to cause pain similar to that seen clinically in normal volunteers, the structure’s susceptibility to painful diseases or injuries and demonstration that the structure can be a source of pain in patients using diagnostic techniques of known reliability and validity [28]. Owing to this definition, lumbar FJ may be implicated in generating low back pain due to their nerve supply, especially in cases of capsular stretching [22]. The fact that pain can originate in the FJ is widely accepted in the literature and is supported by investigations employing articular joint blocks [21]. Meanwhile, some patients may have variations or aberrant innervation of FJ, which may explain false-negative medial branch blocks [9]. Despite technical success, those considerations should be taken into account in patient selection and in FJ denervation procedures. (See below).

## Etiologies of facet joints

### Degenerative process (Fig. 2)

Facet joint degenerative osteoarthritis is the most frequent form of FJ pain, intimately tied to degeneration of the intervertebral discs. Like in all synovial lined joints, osteoarthritis is a continuum between loss of joint space, narrowing, loss of synovial fluid and loss of cartilage and bony overgrowth. High-grade cartilage necrosis arises quite rapidly in FJ. Inflammation generated by degeneration of FJs and surrounding tissues is believed to be a cause of local pain. Prevalence of degenerative FJ is debated in the literature. In a study on 647 cadaveric lumbar spine, Eubanks et al. found that degenerative changes are universal findings with a highest prevalence in L4-L5 spinal level [29]. Evidence of osteoarthritis may be found in early life, with more than one half of adults younger than 30 years and 100% after 60 years, highly suggestive of the major role played by FJs in back pain in the elderly population. In another study, Kalichman et al. showed a high prevalence of FJ osteoarthritis in a community-based population (59.6% of males and 6.7% of females) which increases with age and reaches 89.2% in individuals over 60 years old [3]. Risk factors for lumbar FJ osteoarthritis include: age, sex, spinal level (L4-L5), facet orientation (sagittally oriented) and associated background of intervertebral disc degeneration. This last factor may is often related to the amount of heavy work done before the age of 20. However, the association between degenerative changes in the lumbar FJs and symptomatic low back pain remains unclear and subject of an ongoing debate [3]. Synovial FJ cysts are also associated with radicular pain mimic rather than FJ pain. Indeed, in advanced FJ osteoarthritis, a synovial cyst may appear by a herniation of the synovium through the facet capsule. In contrast to primary facet osteoarthritis, which most often results in low back pain, synovial cysts characteristically cause radiculopathy or symptomatic spinal stenosis by nerve root impingement, particularly in the lateral recesses [30]. Lumbar FJ cysts is associated with higher rates of arthritis and coronally orientated FJ [31]. Lumbar spinal canal or foraminal stenosis may result from degenerative changes in the posterior lumbar spine structures, such as bony proliferation of the FJ themselves (and/or associated with ligamentum flavum thickening) [32].

**Fig. 2:**
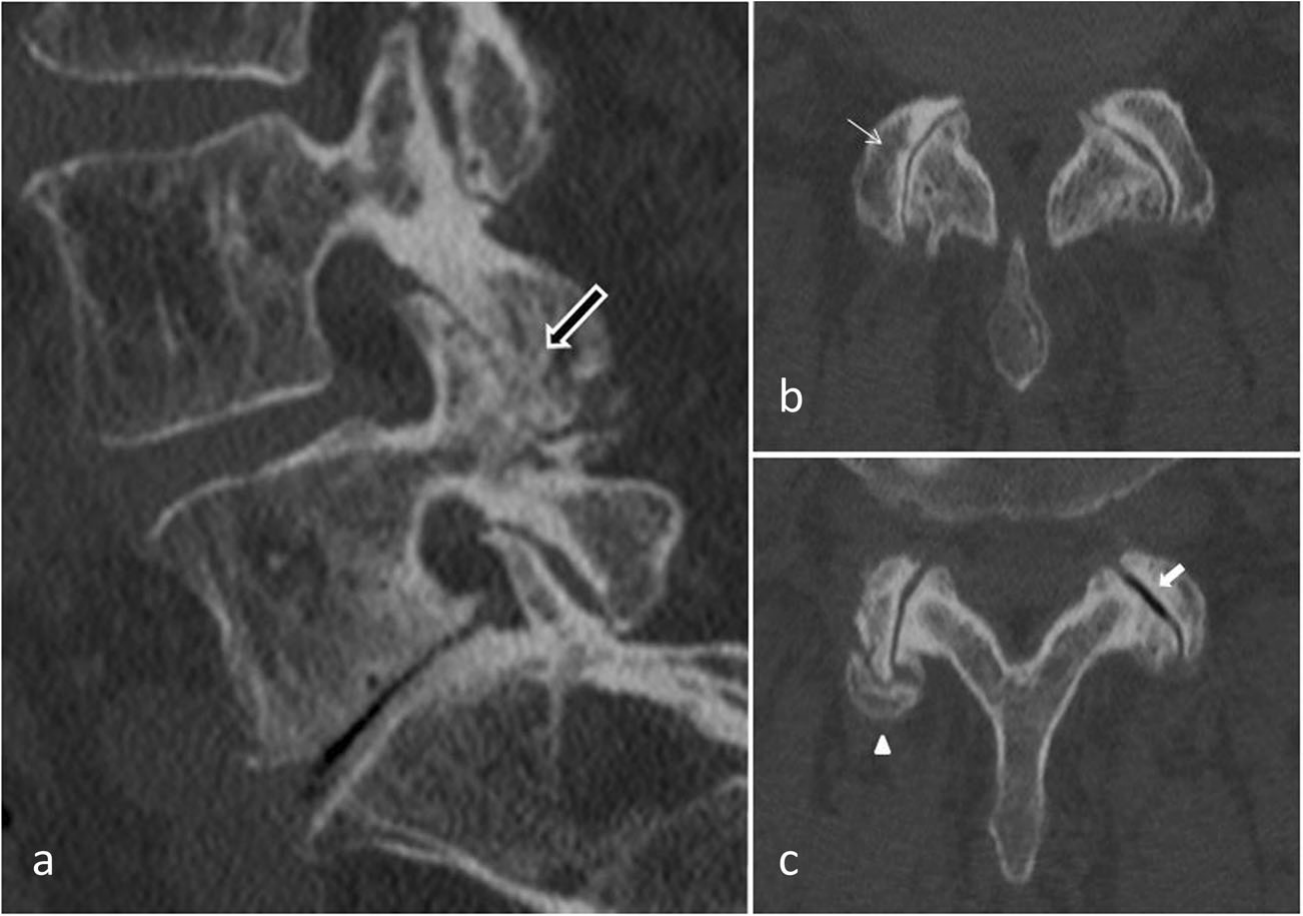
**Degenerative facet joint osteoarthritis (FJOA):** Sagittal (**a**) and axial (**b**, **c**) CT views. Hypertrophy of the posterior articular process (black arrow). Joint space narrowing (thin white arrow). Joint capsule calcification (arrow head) and vacuum phenomenon (white arrow)

### Spondylolisthesis (Fig. 3)

Degenerative spondylolisthesis is the displacement of one vertebra to another in the sagittal plane, which is related in the majority of cases to FJ osteoarthritis and failure of the motion segment. It occurs as a result of subluxation of the FJs, related to an important and progressive loss of cartilage and articular remodelling, with segmental instability causing capsule tension [22]. Spondylolisthesis most often occurs at the L4–5 level, which is predominantly affected by osteoarthritis [33]. In younger populations (30–40 years old), spondylolisthesis can be due to congenital abnormalities, acute or stress-related fractures or isthmic spondylolisthesis. As opposed to its degenerative counterpart, L5–1 is the most affected level, and related instability seems to be more frequent [34].

**Fig. 3:**
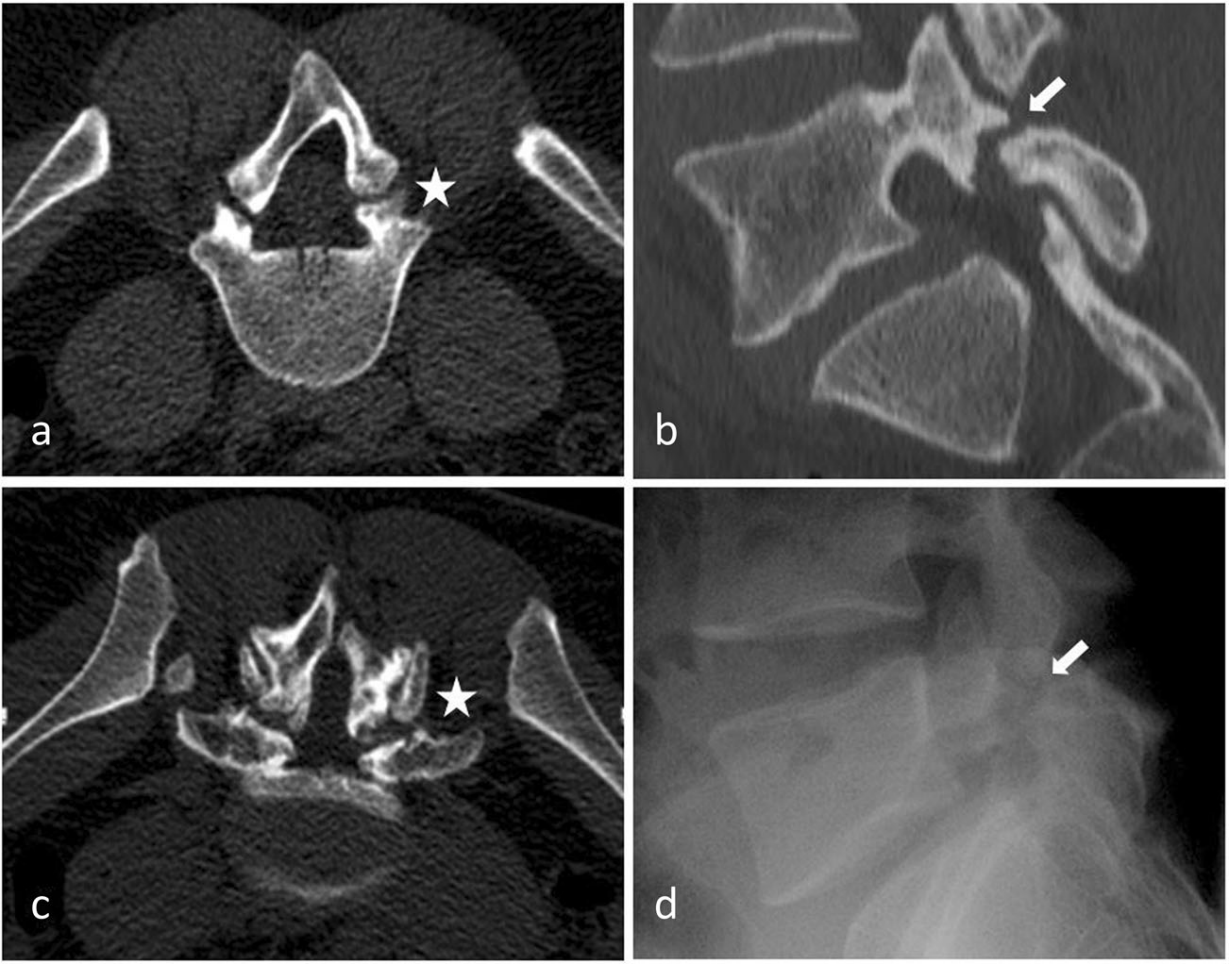
**Isthmic lysis. a**: Axial CT view at L4–5 level; **b**: axial CT view at L5-S1 level **c**: X-ray sagittal view at L5-S1 level; **d**: sagittal CT view L4–5 level

### Septic facet arthritis (Fig. 4)

Septic arthritis is a rare entity [35], which can show similar radiologic findings with more inflammation and a more aggressive signs. It can be secondary to disc or vertebral infection (spondylodiscitis). An isolated form should raise suspicion of tuberculosis or iatrogenic cause. One case of septic arthritis due to *Kingella kingae* has been described [36].

**Fig. 4:**
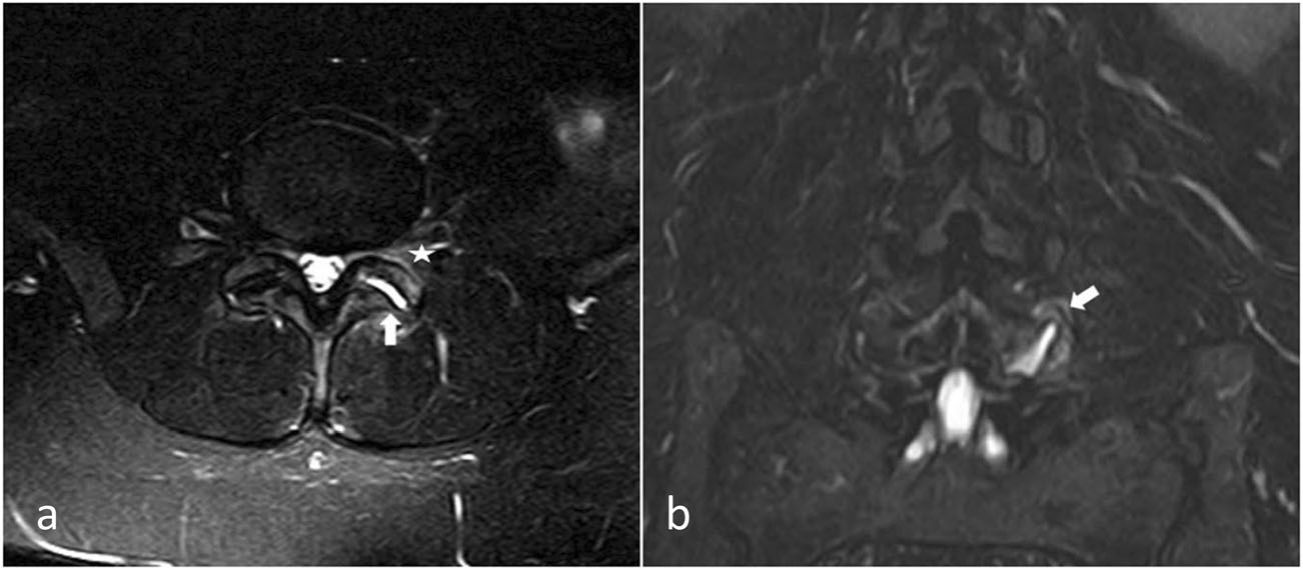
**Septic facet joint arthritis.** Axial (**a**) and coronal (**b**) T2 STIR views. Intra-articular effusion (white arrow) and articular process bone edema (white star). Unilateral signs should raise suspicion of a septic cause

### Inflammatory conditions

Rheumatoid arthritis and ankylosing spondylitis, which are seronegative spondyloarthropathies, may also involve the lumbar FJs, as FJs are synovial joints [18].

## Clinical presentation and pain patterns

**Fig. 5:**
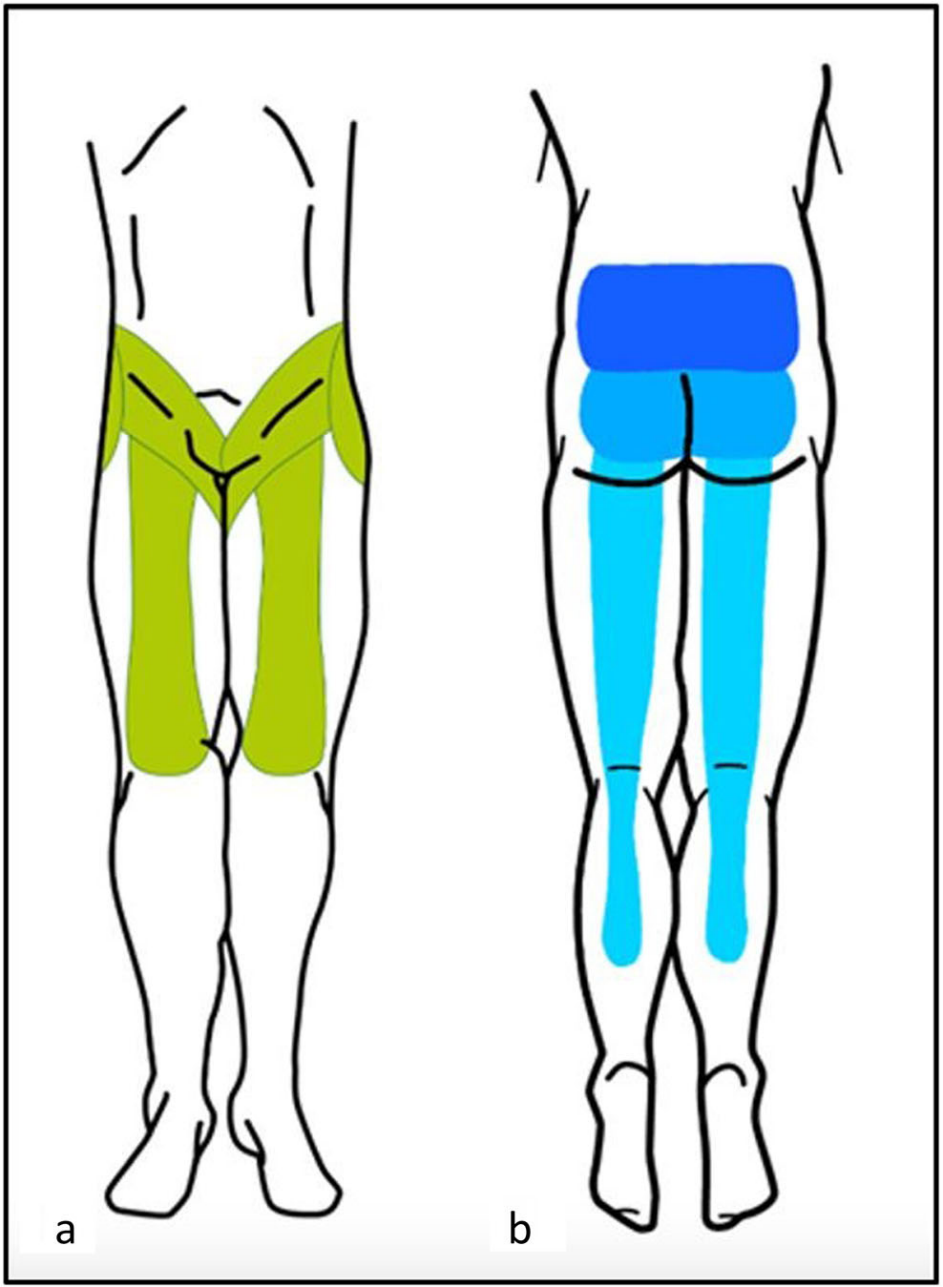
**Facet joint pain radiation**. Posterior aspect of lower limb. Blue: from most frequent (dark blue), to less frequent (light blue) radiating pain areas. Dark blue: pain limited to lower back. Intermediate blue: radiating pain to the posterior aspect of the buttocks. Light blue: radiating pain to the posterior aspect of the lower limbs, may extend lower than the knee level. Green: anterior aspect of lower limb possible radiation areas. **a** anterior aspect of the lower limb (green). **b** posterior aspect of the lower limb etc

FJ as a source of LBP was first described by Goldthwaite in 1911 [37], and Ghormhley who used the term “facet syndrome” to describe a symptom originating from the FJ [38]. It was initially described as lumbosacral pain with or without sciatica in 1933. Ten years later, Badgley et al. suggested FJ as the source of up to 80% of back pain [39]. Facet syndrome included local pain and pseudo radicular radiation with variability of the distribution of referral patterns of pain [3]. Most authors tried to classify the distribution of FJ pain provoked by an infiltration [40] or electrical stimulation [41]. The majority of these studies have not found reliable referral patterns of FJ pain. As suggested by Cohen, this may be explained by the fact that stimulation does not reproduce physiological conditions [8]. FJ pain may be referred distally into the lower limb, thereby mimicking sciatica. “Pseudo-radicular” lumbar pain typically radiates uni- or bilaterally to the buttock and the trochanteric region (from the L4 and L5 levels), the groin and the thighs (from L2 to L5), ending above the knee, without neurological deficits (Fig. 5). However, radiating pain may reach the foot, mimicking sciatic pain, especially in cases of osteophytes or synovial cysts. Claudication is possible. Pain is usually worse in the morning, during periods of inactivity, and following stress exercise, lumbar spine extension or rotary trunk motions, is provoked by standing or sitting positions, and may be elicited on FJ palpation [5]. Pain radiation can be subdivided into primary, secondary and least commonly painful areas as previously described by Barlocher et al. [42]. Abdominal and pelvic pain have also been described [43]. Differential diagnoses include true sciatica, hip pathology (hip osteoarthritis or greater trochanteric bursitis) or sacroiliac impairment. However, lumbar FJ syndrome seems not to be a reliable clinical diagnosis [44], and a specific etiology of back pain can be diagnosed in only about 15% of patients with certainty based on clinical examination alone [45]. The results of studies investigating the FJ as the source of a patient’s symptoms suggest that the currently available tests have limited or no diagnostic validity. Moreover, history and physical examination may suggest but not confirm FJ as the source of pain [6].

## Imaging findings (Table 1)

### X-ray imaging: Radiographs and computed tomography (CT)

The initial radiographic assessment of patients presenting with lumbar facet-mediated pain includes AP, lateral, and oblique views [18]. Oblique radiographs are the best projections for assessing FJs of the lumbar spine because of their oblique position (“Scottie dog”). Lateral films, however, may provide useful information from the isthmus profile such as the pars interarticularis defect. Because of its ability to provide cross-sectional images and to provide a higher contrast between bony structures, CT improves anatomic evaluation of the FJs and is the preferred method for imaging FJ osteoarthritis [46]. However, standard radiographs can also show pathological changes especially in severe disease. Degeneration is characterized by joint space narrowing, sclerosis, subchondral sclerosis and erosions, cartilage thinning, calcification of the joint capsule, hypertrophy of articular processes and of the ligamentum flavum causing impingement of the foramina and osteophytes. Secondary signs include vacuum joint phenomenon (intra-articular gas), joint effusion and associated degenerative spondylolisthesis. Synovial and subchondral cysts can extend posterior to the FJ but also anterior in the spinal cord or neuroforamen. Kalichman et al. showed 24% of X-rays FJOA before 40 years and 89% in the 60–69 years population, but once again with no correlation between abnormal morphology on radiologic findings and back pain [4].

**Table 1:** Main imaging findings in various imaging modalities

X-ray imaging		MRI	SPECT
Radiographs	CT		
AP, lateral (isthmus profile) and oblique views (“Scottie dog”)	Highest contrast between bony structures and adjacent soft tissue	Active synovial inflammation, Adjacent bone edema Fat saturation technique ± Gadolinium injection	99mTc labelled bisphosphonates Osteoblastic activity Hyperemia associated with bone remodelling
Joint space narrowing Subchondral sclerosis and erosions Cartilage thinning Calcification of the joint capsule Hypertrophy of articular processes Vacuum joint phenomenon joint effusion		Facet joint effusion Subchondral bone edema Enhancement of the FJ rim (synovitis) Wraparound bumper osteophyte formation	Increased uptake (nonspecific)

***Associated possible degenerative changes:***

Degeneration of the intervertebral discs

Ligamentum flavum thickening

Degenerative spondylolisthesis (L4-L5 level)

Isthmic spondylolisthesis (L5-S1 level)

Facet joints cysts (coronally orientated FJ)

Lumbar spinal canal or foraminal stenosis

Neural structure impingement

*AP* antero posterior, *CT* computed tomography, *MRI* magnetic resonance imaging, *SPECT* single photon emission tomography

### Magnetic resonance imaging (MRI) (Fig. 6)

MRI is a noninvasive and nonionizing modality that provides excellent soft tissue resolution. The role of MRI in the evaluation of FJ degeneration is not proven. Osteoarthritis may be present in both symptomatic and asymptomatic patients (from 8 to 14%) [47, 48]. Superior sensitivity of MRI compared to CT imaging is controversial [8]. CT and MRI are equally useful in demonstrating morphological changes in FJ. One of the two examinations is thus sufficient for assessing degenerative changes [49]. MRI, however, clearly presents advantages of better assessing the immediate consequences of FJ degeneration, such as surrounding neural structure impingement [50]. Chronic degenerative osteoarthritis processes in these structures involve active synovial inflammation or adjacent bone edema, which can be detected using MRI with a fat saturation technique [51]. Exaggerated fluid in the facets and FJ synovial cysts seen on axial MRI seems to be significantly suggestive of spondylolisthesis and its instability, but is not specific of FJ origin of pain [52]. Recent studies using fat-suppressed MRI sequences have demonstrated that subchondral bone edema is present in the lumbar FJ articular processes in 14 to 41% of patients with back pain [53, 54]. Enhancement of the FJ rim after gadolinium administration will establish a diagnosis of synovitis. Fujiwara et al. proposed a four-grade classification from 1 to 4 [55]: grade 1, normal; grade 2, joint space narrowing or mild osteophyte; grade 3, sclerosis or moderate osteophyte; and grade 4, marked osteophyte. They additionally described the wraparound bumper osteophyte formations which provides an additional stabilizing effect in segmental degenerative disease. An important observation from the Fujiwara study is that MRI tends to underestimate the severity of osteoarthritis of the FJs as compared to CT. The fluid-sensitive sequences on MRI are generally preferred over CT for imaging FJ effusions and juxta-facet cysts; however, they are less sensitive in depicting the joints’ bony cortices and are less accurate in quantifying the amount of sclerosis present. An additional limitation of MRI is that it cannot accurately measure cartilage thinning secondary to the partial volume effect and chemical-shift artefact inherent in this type of imaging. CT is better able to demonstrate the degenerative changes of the FJs because of the high contrast between bony structures and the surrounding soft tissues [18]. However, some authors suggest that TSE T2 fat saturation sequences and, when indicated, gadolinium administration with T1 fat saturation sequences enhance the sensitivity and diagnostic specificity of MR scans. In particular, gadolinium will disclose the active inflammatory stage of a degenerative process thereby identifying new therapeutic targets for percutaneous treatment [51].

**Fig. 6:**
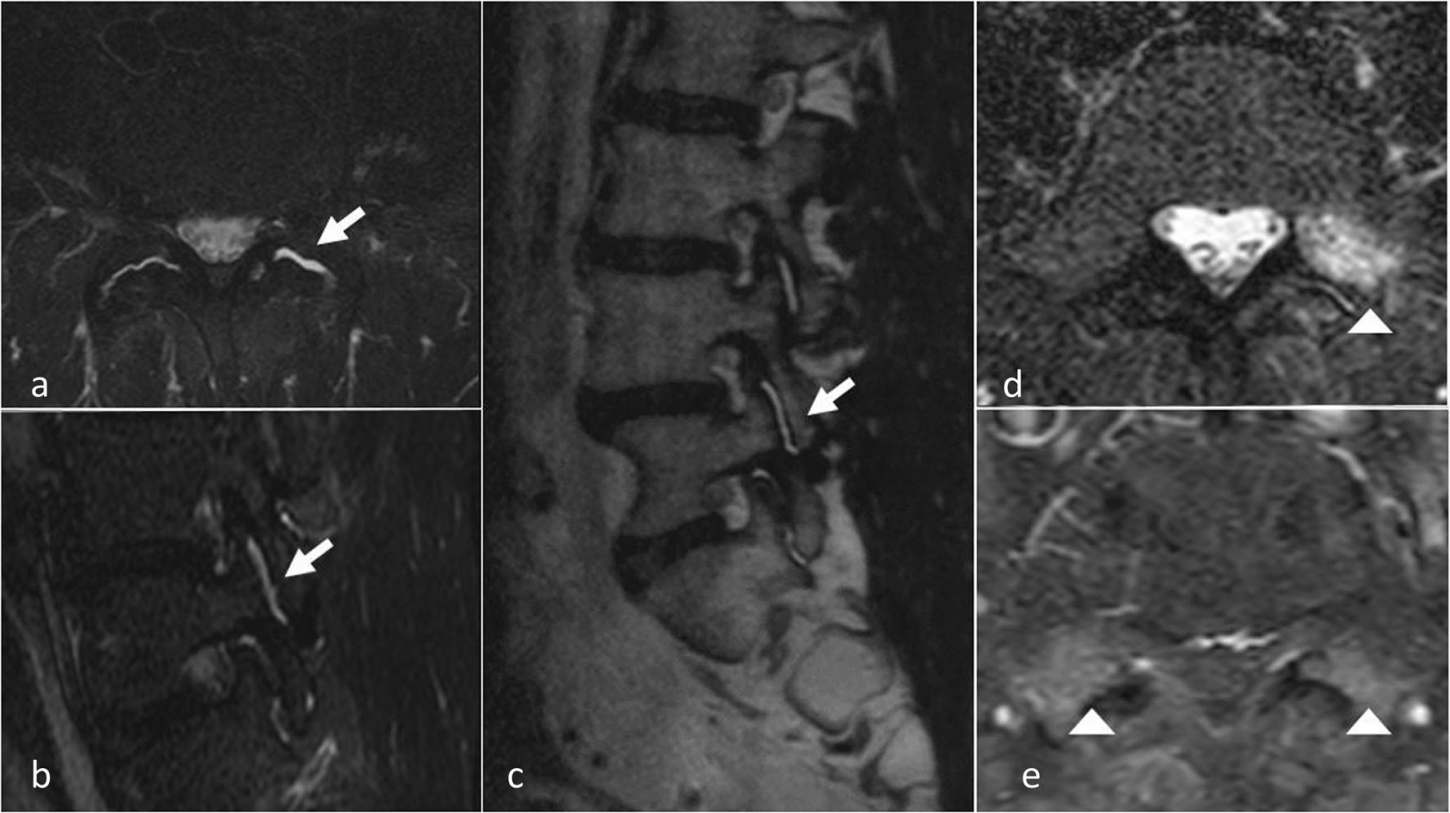
**MRI imaging of facet joints.** Active synovial inflammation and intra articular edema: axial and sagittal T2 STIR views (**a**, **b**) and T2 sagittal view (**c**). T2 STIR and T1 gado axial views (**d**, **e**): articular process bone edema

### Single-photon emission computed tomography (SPECT)

**Fig. 7:**
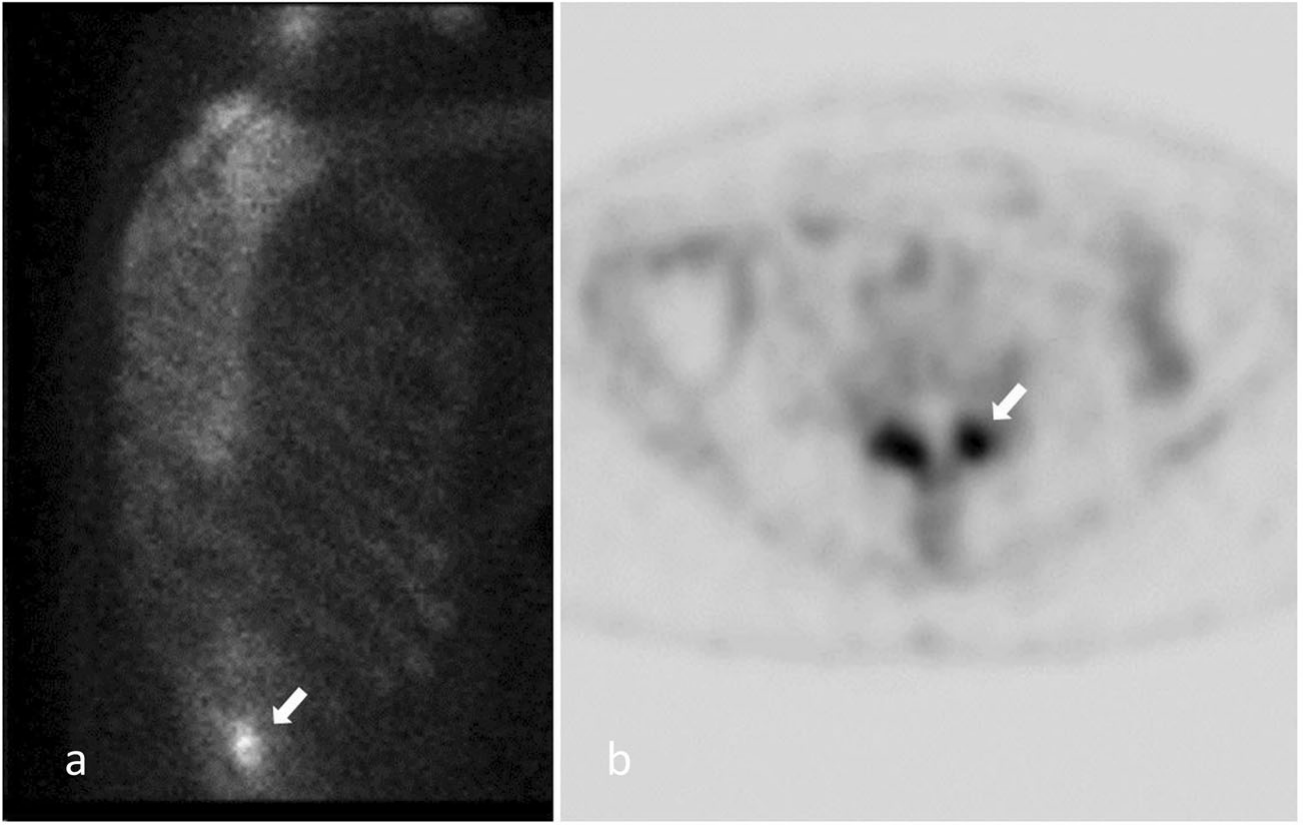
**SPECT imaging of FJ.** Hyperfixation on bone scintigraphy located on FJ capsule inflammation (white arrow)

The detection of FJ inflammation may be more useful than morphological imaging of the joint itself. Radionuclide bone scintigraphy, using 99mTc labelled bisphosphonates, show increased osteoblastic activity along with synovial changes secondary to inflammation or hyperemia associated with bone remodelling (Fig. 7). It has been shown that patients present better improvement after FJ injection in case of positive SPECT findings [56].

### Imaging classification of facet joint osteoarthritis

Two classifications of FJ degeneration are recommended for clinical use. Radiographically, Pathria’s classification classifies FJ arthropathy as well: Facets with joint space narrowing are classified as grade 1, facets with narrowing and sclerosis or hypertrophy as grade 2, and facets with severe degenerative disease encompassing narrowing, sclerosis, and osteophytes as grade 3 [57]. Standard radiographs (Meyerding or Taillard classification) [33] also evaluate motion-related abnormalities in flexion or extension, and assess instability in cases of spondylolisthesis, thanks to dynamic studies. In the setting of degenerative spondylolisthesis, a weight-bearing lateral flexion-extension radiograph is most effective for grading spondylolisthesis and may be needed in addition to MRI and CT imaging. Anteroposterior translation of more than a few millimetres is suggestive of lumbar spine instability in the sagittal plane, which in the appropriate clinical setting may require surgical arthrodesis. In addition to Pathria’s classification, Weishaupt’s grading scheme, based on the agreement between MRI and CT imaging, has been proposed. Facets were again graded from 0 to 3 depending on the degree of joint space narrowing, hypertrophy, sclerosis, and osteophyte formation. The authors recommended against the routine use of CT imaging in the presence of an adequate MRI scan [49]. Fujiwara et al. is credited with developing the standard MRI-based classification system for lumbar FJ osteoarthritis. An additional grading system for foramen stenosis, caused by disc and FJ degeneration can be used as well, based on the depiction of the foraminal components: nerve, vessels and fat [58]. First stage, the non stenotic stage: no modifications depicted. Second stage corresponds to stenosis without evidence of root compression. Third stage, compression of the spinal nerve in the intervertebral foramen caused by either intervertebral disc, flaval ligament or osseous stenosis. In this stage, the content of the foramen is not well identified. A grading scale has also been proposed for lumbar canal stenosis as follows [59]: A) cerebro-spinal fluid (CSF) is clearly visible inside the thecal sac, but its distribution is inhomogeneous. B) Some CSF is still present, giving a grainy appearance to the thecal sac. The rootlets occupy the whole of the dural sac, but they can still be individualized. C) the dural sac demonstrates a homogeneous grey signal with no CSF. No rootlets can be recognized. D) In addition to no rootlets being recognizable, there is no epidural fat posteriorly.

There is currently no consensus on how best to evaluate lumbar FJ osteoarthritis with imaging. It has been reported that in clinical practice, imaging findings of degenerative abnormalities (including radiographs, MRI, CT, SPECT) have been assumed to be associated with nonspecific low back pain [60]. Radiographic changes secondary to osteoarthritis are equally reported among symptomatic and asymptomatic patients. Radiological investigations report a poor correlation between clinical symptoms and degenerative spinal changes [3]. Therefore, the role of FJ imaging in patient with LBP is still debated. The interest of imaging often lies in its ability to rule out differential diagnosis, commonly referred to as “red flag indications”, rather than to prove a symptomatic condition. Red flag indications are intended to represent the potential for life or limb threatening conditions (suspicion of aortic aneurysm or dissection, neoplasm, infection, cauda equina syndrome, fracture, motor weakness). Advanced diagnostic imaging of the symptomatic level is appropriate and/or work-up for a non-spinal source of spine pain [61].

## Interventional management

First-line therapy consists in conservative multimodal management such as pain medication (acetaminophen, nonsteroidal anti-inflammatory drugs, muscle relaxants, antidepressants), physiotherapy, acupuncture, and, if necessary, psychotherapy [8].

As mentioned above, because radio-clinical correlation is not reliable in patients with LBP, the diagnostic and therapeutic role of interventional procedures targeting the FJ have been reported in chronic spinal pain in patients who have failed conservative management [62]. Whatever the technique used, it has been shown that the physician’s attitude seems to affect the clinical outcome of a procedure by a hetero-suggestion phenomenon, with better results [63]. Imaging guidance has shown to both to increase technical and clinical efficacy and reduce potential complications [64]. Common complications of FJ procedures include: hemorrhagic, infectious complications, and vasovagal syncope [65].

### Blocks

Because no clinical features or diagnostic imaging studies can determine whether an FJ is painful or not, controlled blocks are the only reliable tool in the diagnosis of FJ pain as a cause of LBP [7]. Diagnostic blocks of nervous structures that are suspected to generate pain can be performed to evaluate the role of the target structure in the painful syndrome [9]. However, several debates exist on the technique and the definition of the performed block:

#### The degree of the relief that should occur

Bogduk defined specific criteria for an optimal selection as an anatomically accurate block under guidance with ideally complete relief of pain following an MBDR block. Manchikanti et al. defined at least an 80% reduction of pain and the ability to perform previously painful movements [66]. More liberal criteria have also been reported, such as greater than 50% relief of pain [9].

#### The target of the block (Fig. 8)

The first results comparing intra-articular and medial branch block have reported similar outcomes [67, 68]. However, a recent review showed higher evidence in short- and long-term relief with medial branch blocks versus intra-articular blocks [2]. Moreover, intra-articular blocks appear less anatomically accurate and have not been validated as predictive of response to any form of treatment [9]. Medial branch block also seem to present higher specificity to select patients for medial branch neurolysis [69]. Moreover, it seems technically easier to perform using anatomic landmarks [70], than intra-articular injections, providing the use of MDBR block in patient selection before denervation procedures [8].

**Fig. 8:**
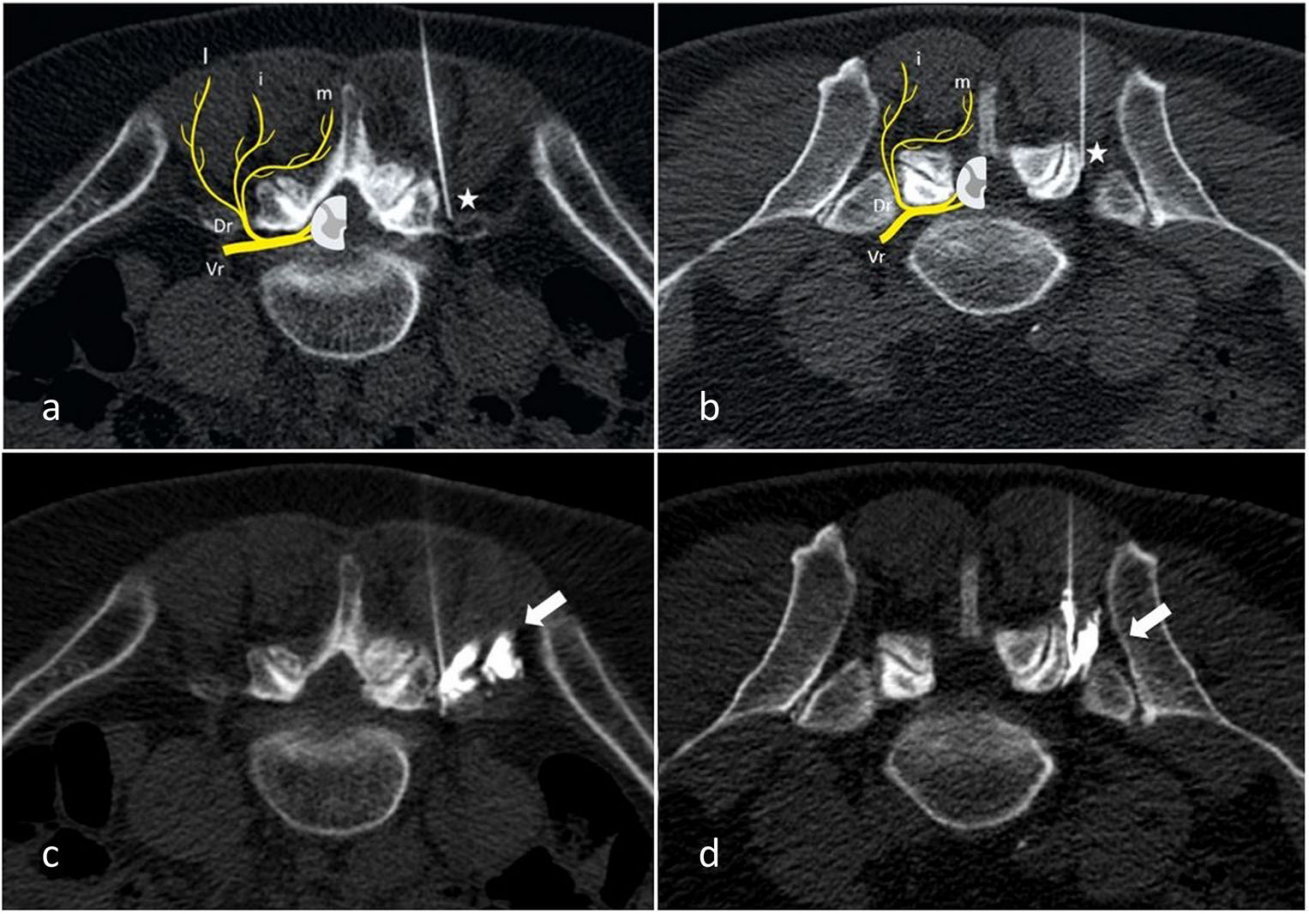
**Medial branch block under CT guidance. a**: L4–5 level; **b**: L5-S1 level. **c**, **d**: Diffusion of contrast media prior to anesthetic injection confirming optimal needle tip placement (white arrows). Needle tip at the target point at injection should be placed at the middle of the base of the transverse process at its junction with the superior process at the L4–5 level. An analogous target point should be used at the L5-S1 level midway between the upper end and middle of the ala of the sacrum (white stars). Vr: ventral ramus. Dr: Dorsal ramus. m: medial branch. i: intermediate branch. l: lateral branch

#### The number of blocks and the levels which should be targeted

A definitive diagnosis of FJ mediated pain may require blocks at two separate sessions. When performing a single-level block only, there is a high false-positive rate (30–45%). Some authors have therefore advocated the performance of repeated blocks [71]. Cohen et al. showed a success rate of lumbar FJ radiofrequency (RF) denervation patients of 39% after a single block and 64% after a double block [72]. Because of the dual nerve supply of FJs, at the same level and the level above, diagnostic blocks should be performed with a minimum of two levels to block a single joint [66].

#### Injected drugs

Diagnostic blocks commonly include local anesthesia (lidocaine and/or bupivacaine) with or without steroids injections [8]. Some find an advantage of adding steroid injection [66] (see below).

### Steroid injections

In the majority of the reported studies, FJ injection include long acting corticosteroids (anti-inflammatory and antiedematous effect, immunosuppressive action and inhibition of neural transmission within the C fibres) and local anesthetics [8]. FJ can be infiltrated with intra-articular, periarticular and medial branch injections. Due to the presence of inflammatory mediators into and around degenerative FJ, short- to intermediate-term pain relief should occur after steroids injections. However, discrepancies persist in the literature about the efficacy of steroids for FJ pain [8]. Although intra-articular injections (with or without steroids) have traditionally been used in the diagnosis of FJ pain, a controlled trial by Lilius et al. reported no outcome differences between intra- and periarticular injections [73]. European guidelines do not recommend the use of intra-articular steroids in management of chronic LBP [74].

### Neurolysis

The ideal candidate for FJ denervation is a patient who underwent medial branch infiltration with significant pain relief after failure of conservative management. Because of the dual nerve supply of a given FJ, electrodes or cryoprobes should be placed at two subsequent levels [41]. Nerve fibres can either be destroyed by physical means: heat (radiofrequency) or cold (cryoneurolysis), or by chemical means (alcohol/phenol). The main characteristics of these techniques are described in Table 2. Lumbar medial branch neurolysis achieves relief of pain, improvement in disability, and reduction of the need for analgesics [9]. Whatever the technique used, neurolysis does not allow definite pain relief. The destroyed nerve will eventually regenerate, and in consequence, recurrence of pain may occur. The procedure can be repeated [9]. Currently, the two most widely reported techniques are radiofrequency (RF) and cryoneurolysis (CN). In both techniques, before injection of local anesthetics and thermal lesions, electrical stimulation monitoring should be performed to ensure safety in performing thermal denervation [75]. Currently, ISIS recommends a maximum of two FJ denervation per year [76]. Although RF techniques have been described in detail with a possibly longer effect than CN, in our experience it may appear as a slightly more challenging technique.

**Table 2:** Main characteristics of the denervation procedure

Radiofrequency	Cryoneurolysis		Chemical neurolysis
Principle	Sinusoidal current Ionic agitation Tissular heating by friction T > 45 C°	Joule–Thompson effect Decompression of CO2 or N20 Ice ball T > −20 C°	Protein denaturation
Advantages	Possibly longer effect Technique described in more detail Abundant literature Wider range of needles available	Neuroma Neuritis Less tissue damage Technically easier (bigger lesion)	Cheap Available
Disadvantages	Neuroma formation(rare) Neuritis More tissue damage Technically more challenging	Duration of effectiveness less assessed Larger probes and coaxial needles	Not widely used in this indication Neuritis Neuroma Tissue necrosis Deafferentation pain Uncontrolled diffusion

### Physical neurolysis

#### Radiofrequency ablation (RFA) (Fig. 9)

##### Principle

RF consists in the placement of electrodes under imaging guidance, delivering a sinusoidal current (400–500 kHz). Regions crossed by the current undergo an ionic agitation which leads, through particle friction, to tissular heating. The sought purpose is to expose nerve cells to a temperature > 45 °C causing an irreversible cellular denaturation [77]. A wide temperature range (70–90 °C) has been reported in the literature with good results [78]. Another possibility is the use of pulsed RF (application of RF energy with pulsed time cycles at temperatures not exceeding 42 °C). The rationale for the use of pulsed RF is to avoid any potential inadvertent damage to adjacent nerve roots as well as possible secondary spinal instability due to muscle denervation [14]. However, the use of this technique appears to be less effective in the long term [79]. Therefore, pulsed RF does not appear as a substitute for conventional thermal lumbar medial branch neurotomy [9].

**Fig. 9:**
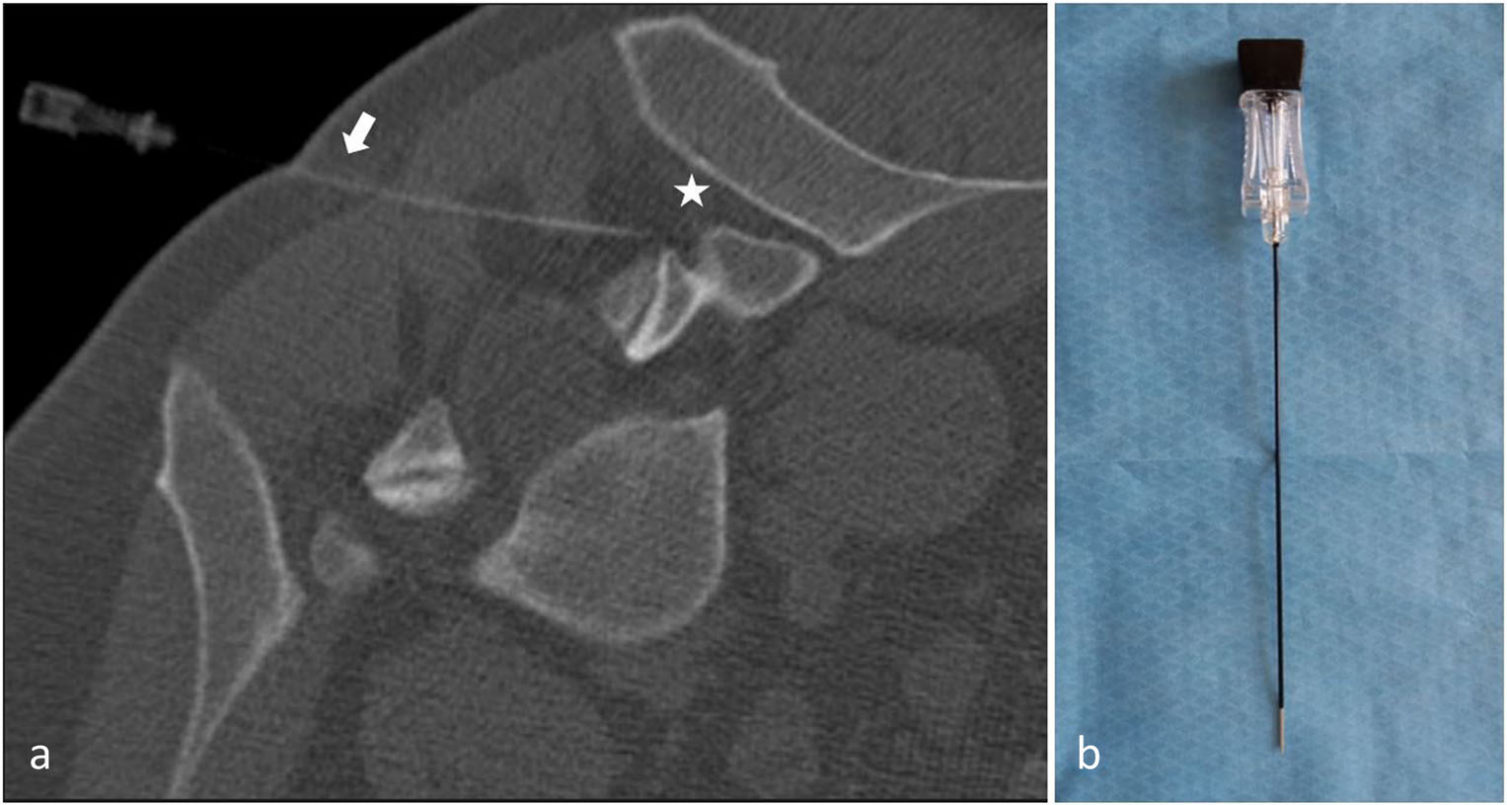
**Facet joint radiofrequency ablation. a**: Radiofrequency ablation at the right L5-S1 level. Appropriate electrode placement (white arrow) parallel to the target nerve (white star) in order to achieve denervation along a substantial segment of the targeted nerve. **b**: 22G Radiofrequency needle showing uninsulated tip

##### Technique

Bogduck et al. underlined the importance of patient selection and the use of a properly performed technique [80]. Appropriate technique is described in the ISIS guidelines [81] where emphasis is made on the electrode placement: parallel to the target nerve in order to achieve denervation along a substantial length of the targeted nerve [26]. These considerations seem more important to take into account with the RF technique than with CN, where circumferential lesions are less extensive than with cryoprobe [82]. RF probes produce transverse lesions around the electrodes, but little lesioning at the needle tip. Perpendicular placement may miss the targeted nerve [80]. Moreover, operators should not rely on single placement of the electrode, and multiple placements may be required in order to cover all possible variations of the nerve [9].

##### Results

In a prospective study, Dreyfuss et al. showed that under these conditions some 60% of patients could expect at least a 90% reduction in pain, and 87% could expect at least 60% reduction lasting 12 months [77]. In Kessinger et al.’s study, conducted in patients with minor degenerative spondylolisthesis, 60% of patients sustained at least 80% pain relief lasting at least 12 months; 80% sustained at least 60% relief [83]. Several controlled studies confirmed this trend [69, 84–87], with a mean decrease of 2–3 points on a visual analogue scale vs control groups. RF complications are uncommon (1% incidence), of limited duration and minor in nature [88]. Potential side effects include painful cutaneous dysesthesias or hyperesthesia increased pain due to neuritis, neuroma formation, and deafferentation pain. Unintentional damage to a spinal nerve causing a motor deficit, is also a complication [89]. Sensory and motor stimulation during the procedure may help to avoid this complication [75].

#### Cryoneurolysis (CN) (or Cryoneuroablation or Cryoanalgesia) (Fig. 10)

##### Principle

Cryoneurolysis is an application of cold to the nerve to cause its denaturation. The physical principles relies on the Joule–Thompson effect, which is based on a rapid decompression of gas (either N_2_O or CO_2_) at the extremity of the probe, capable of delivering ice-cold temperatures of up to −70 °C [75]. At the tip of the needle, an ice ball is created in the surrounding tissues. It induces a conduction block, similar to the effect of local anesthetics (all nerves fibres stop conducting at −20 C°). Long-term pain relief from nerve freezing is obtained because of the vascular damage caused by ice crystals to the vasa vasorum, which causes endo-neural edema and cell death.

**Fig. 10:**
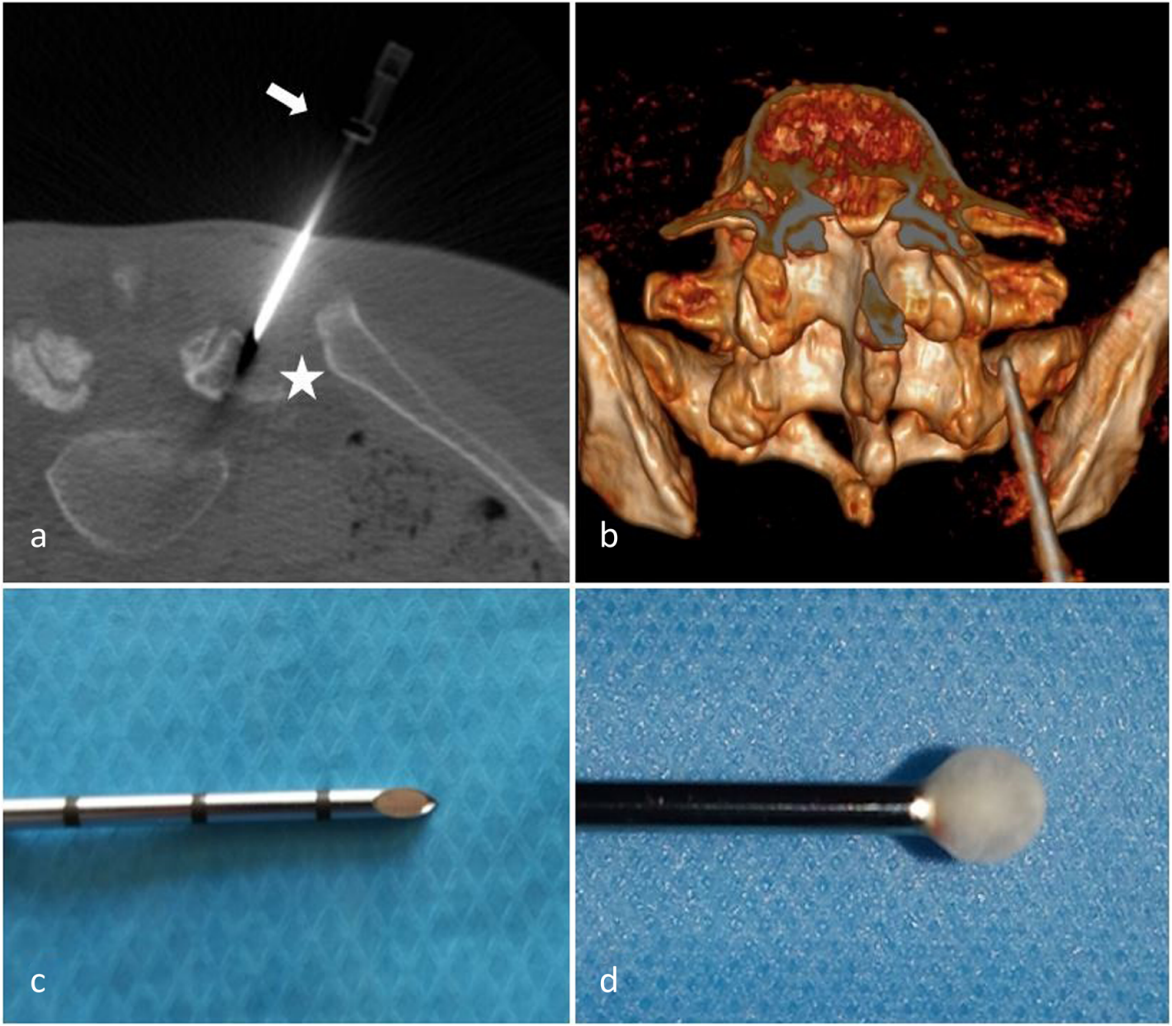
**Facet joint cryoneurolysis.** CT-guided cryoablation at the right L5-S1 level. **a**: Anatomically accurate cryoprobe (white arrow) placement with the target midway between the upper end and middle of the ala of the sacrum (white star). **b**: 3D reconstruction showing the same cryoprobe placement. Cryoprobe needle tip (**c**): ice-ball formation at the cryoprobe tip (Joule–Thompson effect) (**d**)

##### Technique

As with RFA, the success of cryoneurolysis is dependent on patient selection and accurate probe placement, which should follow the same guidelines described by the ISIS. The extent and duration of the effect is therefore a function of the degree of cold obtained and the length of cold application [73]. In contrast to RFA, a tangential approach of the probe is not essential [90]. Minimal, if any, sedation should be used, as the patient must be conscious to respond to sensory and motor stimulation [75]. Moreover, intra-procedural pain in CN appears to be tolerable [91].

##### Results

Lloyd proposed CN as superior to chemical neurolysis [90]. However, CN technique has been described as less accurate than RF. The lasting effect compared to RF also seems unclear. No studies comparing CN and RFA in FJ pain management are available to date. Three recent prospective studies [42, 92, 93] showed a reduction of pain at 6 weeks and 3 and 6 months, with a 50% pain decrease. A recent retrospective study by Wölter et al. in 2011 confirmed this trend [91]. Advantages of CN include less tissue damage, less risk of neuroma or neuritis, and a larger denervation area at the needle tip [91, 94].

### Chemical Neurolysis

This technique requires the administration of a chemical agent able to destroy neural structures (protein denaturation) [95] involved in the perception of pain to promote long lasting analgesia. The size of the lesions varies according to the concentration, and therefore the quantity. The two neurolytic agents most widely used in the treatment of chronic pain are phenol and alcohol, producing a block that lasts 3–6 months [96]. Major drawbacks with the use of these agents include: necrosis of surrounding tissue, neuritis, and uncontrolled diffusion (83). Furthermore, these powerful neurolytic agents may induce sequelae in the axonal membrane, which might explain cases of painful paresthesia observed several months following a neurolytic block: this is known to be deafferentation pain sequelae [97]. These techniques are also associated with neuroma formation [98].

#### Alcohol

The neurolytic effects of ethyl alcohol at a concentration greater than 50% are well known, but higher concentrations (95–100%) are required for nerve destruction to be permanent [99]. Alcohol is extremely irritating to both neural structures and surrounding tissues, causing pain, burns, and local hypersensitivity. Alcohol neurolysis usually causes severe, intense pain, which quickly disappears. Alcohol is associated with a higher rate of neuritis than phenol [100].

#### Phenol

As with alcohol, neurolysis depends on the concentration used: the efficacy of 3% phenol in saline is comparable to that of 40% alcohol. Phenol is responsible for a transient local anesthetic effect (between 5 and 20 weeks). Aqueous phenol is easy to use, with a low potential of diffusion, and does not cause violent pain on injection [99].

### Facet joint denervation: How to do it?

#### Selection processing

The following criteria should be noted prior to the procedure: history of back pain surgery, description and radiation of pain, mean duration of pain, pain intensity on a numerical pain scale (0–10). Despite the lack of specificity, a physical examination should be performed. Prior imaging studies should be analysed and red flags should be ruled out.

#### Level

As physical and neurologic examination do not identify symptomatic FJs, and structural findings of FJ osteoarthritis on imaging are not predictive of FJ pain origin, FJs targeted for blocks are chosen based on a combination of clinical and imaging data. Indeed, FJ level can be deduced by comparing the patient’s pain to FJ pain referral maps, T2WI MRI hyperintensity, gadolinium enhancement or increased uptake on SPECT, which may help to identify painful joints.

#### Block

This procedure can be done under fluoroscopic or CT guidance. Our practice is to use CT guidance and medial branch block. The patient is placed in prone position. An initial, non-enhanced planning CT is performed from the subsequent level in order to determine target and the safest needle pathways. The skin entry point is marked, and a local skin scrub is performed. Needle progression (22G) is performed on axial view under CT guidance (oblique view in case of fluoroscopic guidance) until the needle tip artefact is located at the defined target (at the same level and the level above). The tip of the needle should be placed in the angle formed by the transverse process and at the neck of the medial aspect of the superior articular process in case of L1–4 level or midway between the upper end and middle of the ala of the sacrum at L5–1 level. Diluted iodinated contrast is injected (1 mL) in order to control accurate needle positioning. A mixture of fast and slow acting anesthetic (1 mL mixture of lidocaine hydrochloride 1%, and of ropivacaine hydrochloride 2 mg/mL). Patients are then asked to report pain relief in the following 12 h, both by self-reported improvement (percentage of pain decrease) and VAS score.

#### Neurolysis

**Fig. 11:**
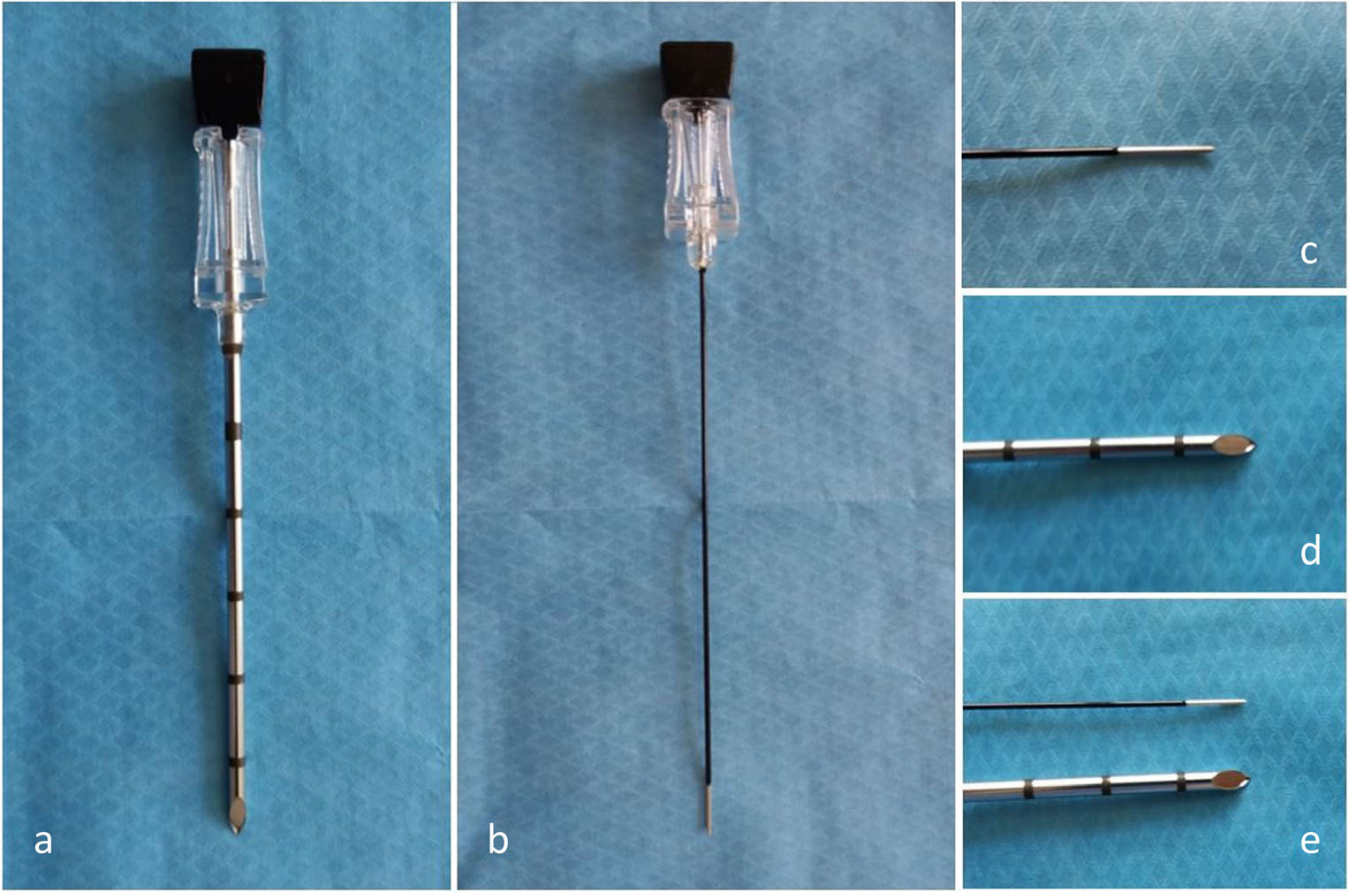
**Photographs of the coaxial needles:** for cryodenervation (**a**, **d**) and radiofrequency (**b**, **c**), highlighting the difference in diameter 12G vs 22G (**e**)

As with block tests, this procedure can be done under fluoroscopic or CT guidance. A 22G cannula (RFA) or a 12G insertion cannula (cryoneurolysis) is inserted as previously described (Fig. 11). The stimulation mode is the crucial step: a sensitive stimulation (frequency 50-100 Hz) is first performed, which should produce a tingling sensation in the painful area. Motor stimulation (frequency 2-5 Hz) is then performed and should not provoke leg muscle contraction. Caution is necessary in the case of sedation, as the stimulation threshold is biased by the neuroleptanalgesia. In the case of RFA, one to three cycles (90 s) between 70 and 90 °C may be performed, with slight needle repositioning between each cycle. Local anesthesia may be needed in case of pain during the heating process. A steroid injection may be added to avoid secondary neuritis. In the case of cryoneurolysis local anesthesia is generally not necessary. One to two cycles may performed.

#### Follow-up

All patients should be followed up by physicians in the interventional radiology unit to assess the efficacy and possible complications with the same criteria. Because of a high false-positive rate, in cases of primary block test failure, this test should be repeated before any denervation procedure. Repeated infiltrations may be proposed in case of contraindications or refusal of denervation procedure. Neurolysis may also be repeated.

## Surgical management

The results of FJ blocks to predict lumbar surgical outcomes and surgical therapies including arthrodesis for degenerative FJ disorders are discouraging [8]. There is no convincing evidence to support any surgical intervention for FJ degenerative pain. In case of spondylolisthesis, pain relief may be obtained with arthrodesis when interventional management fails, but there are currently no guidelines available. In most cases, non-operative treatment should be attempted before surgical management. Some suggest that the optimal surgical management is a decompressive lumbar laminectomy in patients with grade I or II. On occasion, for those with foraminal/far lateral pathology at the level of the listhesis, patients may require additional non-instrumented or instrumented lumbar fusions [101]. Although there currently is no consensus, FJ neurolysis may be used as a therapeutic tool in cases of surgical management failure on low back pain relief.

## Other interventional treatments

Other more recent techniques or imaging guidance have been described in the literature, but will need further assessment. Wu et al. recently compared the effectiveness and safety between autologous platelet-rich plasma (PRP) and local anesthesia/corticosteroid in intra-articular injection for the treatment of FJ syndrome. They showed that prone autologous PRP presented a superior efficacy with longer duration [102]. An observational retrospective study of 86 patients by Kirchner et al. confirmed this trend [103].

Iwatsuki et al. performed laser radiation of the dorsal surface of the facet capsule in 21 patients and reported greater than 70% pain relief for at least 1 year in 81% (17 patients) [104]. Feasibility and safety of MRI-guided focused ultrasound ablation of the lumbar medial branch nerve has been shown in a swine model and thermal necrosis was confirmed [105].

## Conclusion

Because chronic low back pain of facet joint pain origin represents a major health care problem, diagnosis and management of such a high prevalent condition as facet joint syndrome is a major socioeconomic burden. Because of the ability of facet joint pathology to mimic spine root compression, the low specificity of FJ syndrome and inefficient use of lumbar imaging, it appears as a misunderstood, misdiagnosed and improperly treated pathology. Facet joint-related anatomical, clinical and radiologic knowledge is essential for successful facet joint syndrome management. Diagnostic blocks are a keystone of facet syndrome diagnosis. If diagnostic blocks of the nerves that supply specific facet joints relieve the patient’s pain, denervation procedure lesioning of the same nerves can be offered to provide prolonged benefit. The role of the radiologist is essential in the management of these patients, and radiologists should embrace all aspects of facet joint pain management, from diagnosis—enabled by high-performance modalities available—to interventional management. The radiologist can therefore play an active role in the difficult task of alleviating patients’ chronic low back pain of facet joint origin.

## Abbreviations

CN: Cryoneurolysis
CT: Computed tomography
FJ: Facet joint
LBP: Low back pain
MAL: Mamillo-accessory ligament
MBDR: Medial branch of the dorsal rami
MRI: Magnetic resonance imaging
RF: Radiofrequency
RFA: Radiofrequency ablation
SAP: Superior articular process
SPECT: Single-photon emission computed tomography
